# Author Response: Local Geographic Atrophy Growth Rates Not Influenced by Close Proximity to Non-Exudative Type 1 Macular Neovascularization

**DOI:** 10.1167/iovs.63.5.11

**Published:** 2022-05-10

**Authors:** Omer Trivizki, Eric Michael Moult, Liang Wang, Prashanth Iyer, Yingying Shi, Giovanni Gregori, William Feuer, James G. Fujimoto, Philip J. Rosenfeld

**Affiliations:** 1 Department of Ophthalmology, Bascom Palmer Eye Institute, University of Miami Miller School of Medicine, Miami, Florida, United States; 2 Department of Electrical Engineering and Computer Science, Research Laboratory of Electronics, Massachusetts Institute of Technology, Cambridge, Massachusetts, United States. E-mail: prosenfeld@miami.edu.

We thank Pfau et al. for their thoughtful letter regarding our recent article.^1^ Their letter raises several interesting and important points, which we address below.

Pfau et al. are correct in writing that we computed *P* values for each eye separately and that we did not condition our analyses on geographic atrophy (GA) growth rates from natural history studies or fellow eyes. However, the hypothesis tested in our paper pertains to local (i.e. spatially resolved) relationships, and therefore is inherently intra-eye. In particular, our hypothesis involves comparing growth rates along different segments of the same GA lesion, making (global) growth rates of lesions in other eyes not relevant to the hypothesis under consideration in our paper. Note that the “spatially specific hypothesis,” as defined in their letter, is also intra-eye. The corresponding inter-eye (population level) hypothesis is that global (i.e. spatially unresolved) GA growth rates are reduced by the presence of non-exudative type 1 macular neovascularization (MNV). Although the global hypothesis is certainly of interest, it was not a focus of our study.

We broadly agree with Pfau et al. that there is merit to distinguishing the spatially specific hypothesis from the “halo hypothesis.” However, we consider these two hypotheses to represent a difference in degree rather than in kind, with the former a limiting case of the latter—namely, if the “halo” is made very small, one naturally arrives at the spatially specific hypothesis. Thus, the relevant question pertains to the most appropriate size of the halo. Although we remain uncommitted on this question, we do briefly mention some possibilities in the Discussion portion of our paper using the term “neighborhood-of-influence” rather than halo, and we partially address it by considering both an all-distances halo and a 1 mm halo. Indeed, we believe that our analysis is well suited to detecting effects across the range of plausible halo sizes—including the spatially specific limit. For example, if the spatially specific hypothesis were true, we would expect to see a sharp jump in the scatter plots of the local growth GA growth rates versus distances-to-MNV. However, such a trend was not present (Figures 3–5).^1^

We have some disagreement with Pfau et al. regarding their interpretation of Figures 3–5 of our paper. Specifically, Pfau et al. wrote that these figures “…reveal a slower median RPE atrophy progression in the subregion co-localizing with MNV (0 to 0.1 mm subregion) compared to the overall median elsewhere (patients 1, 4, 5, 7, 8, and 9 [i.e. 6 of 9 patients])…”. However, in case 8, the configuration of the MNV and GA is such that there are no GA margin points—at all—in the 0 to 0.1 mm range, and so case 8 can neither support nor refute the spatially specific hypothesis. Furthermore, in case 5, the median growth rate in the 0 to 0.1 mm range is higher than that in the adjacent 0.1 to 0.2 mm and 0.2 to 0.3 mm ranges. Because points in the neighboring ranges would be the most natural “controls” for the spatially specific hypothesis, but not, incidentally for the halo hypothesis, case 5 also does not support the spatially specific hypothesis. This reduces the tally to four of nine patients. Finally, there is the question of statistical significance. Because GA growth rates have a substantial spatial autocorrelation—that is, nearby margin segments tend to have similar growth rates—it would not be unlikely that by sheer chance the margin segments in the 0.1 to 0.2 mm range enlarge slower (or faster) than margin segments farther from the MNV, in some cases. We believe that accounting for this autocorrelation is important for reducing type I error rates.

Finally, we want to emphasize our belief that atrophy embedded within MNV, most likely arising from a pre-existing MNV, is of a different etiology and character than that of GA that independently developed,^2^^,^^3^ and, consequently, that the subjects of our study may not be directly comparable to those of the study by Pfau et al.^4^

In conclusion, we thank Pfau and colleagues for their interest and engagement in this exciting topic and believe that they raise important points, particularly regarding the distances over which MNV lesions may influence GA growth rates. Although not addressed by our study, we also believe that the effects of MNV on global GA growth rates is an important topic for future investigations. Finally, we echo their letter in emphasizing the need for larger prospective studies.
